# DESIGN AND IMPLEMENTATION OF THE ACHIEVE SUCCESSFUL AGING HEALTH EDUCATION CONTROL INTERVENTION

**DOI:** 10.1093/geroni/igad104.0298

**Published:** 2023-12-21

**Authors:** Nancy Glynn, Theresa Gmelin

**Affiliations:** University of Pittsburgh School of Public Health, Pittsburgh, Pennsylvania, United States; University of Pittsburgh, Pittsburgh, Pennsylvania, United States

## Abstract

Choice of comparison group was essential for participant recruitment and retention for the ACHIEVE Study and necessary to control for key components of the hearing intervention. The Successful Aging (SA) Health Education control intervention followed the protocol and materials developed for the 10 Keys to Healthy Aging (10Keys™), an evidence-based interactive health education program for older adults on topics relevant to chronic disease and disability prevention, previously implemented in other trials. The 10Keys contained up-to-date prevention guidelines based on the current recommendations from leading groups (e.g., United States Preventive Services Task Force, Centers for Disease Control and Prevention). To control for general levels of staff and participant time and attention, participants randomized to the SA control met individually with a 10Keys certified health educator who administered the program every 1-3 weeks, totaling four visits over ∼2-3 months. To match the contact schedule with the hearing intervention and promote retention, participants returned for booster sessions semi-annually. During COVID-19 restrictions, we delivered booster sessions by phone. Individually tailored session content included a standardized didactic education component (a 10Keys module) as well as activities, goal-setting, and optional extracurricular enrichment. To further enhance retention and perceived benefit, each session also included a 5- to 10-minute upper body extremity stretching program. We offered the SA control participants the hearing intervention and vice versa at the trial conclusion. Monthly committee calls ensured fidelity and allowed for troubleshooting any participant-related issues. Audio recordings of sessions delivered by health educators were reviewed monthly for quality control.

